# Editorial: Neural Signal Estimation in the Human Brain

**DOI:** 10.3389/fnins.2016.00185

**Published:** 2016-04-29

**Authors:** Christopher W. Tyler, Clare Howarth, Lora T. Likova

**Affiliations:** ^1^Division of Optometry and Vision Sciences, Centre for Applied Vision ResearchCity University London, London, UK; ^2^Smith-Kettlewell Eye Research InstituteSan Francisco, CA, USA; ^3^Department of Psychology, University of SheffieldSheffield, UK

**Keywords:** human brain, neural signal estimation, fMRI, MEG, EEG

The ultimate goal of functional brain imaging is to estimate the neural signals that flow through the brain, mediating behavior, and conscious experience during the spectrum of activities controlled by the nervous system. Although, various brain imaging techniques are in routine use, determining the underlying neural activity remains a challenge (Lopez da Silva, 2010). Despite its impressive spatial resolution, functional Magnetic Resonance Imaging (fMRI) measures a blood-oxygenation-level-dependent (BOLD) signal and, hence, only indirectly reflects the nearby neural activity. The interpretation of these signals is further complicated as it is sometimes unclear what aspects of “neural activity” BOLD represents. “Neural activity” could refer to spiking activity, subthreshold activation, or synaptic currents, each of both excitatory and inhibitory neurons, to name a few. Although, early findings suggested that BOLD was directly proportional to average neuronal firing rates (Heeger et al., 2000; Rees et al., 2000), in the cortex BOLD fMRI signals are marginally better correlated with LFPs (reflecting slow waveforms of neural activity) than with MUAs (reflecting spiking; Logothetis et al., 2001), suggesting that they may preferentially reflect inputs and intracortical processing (Viswanathan and Freeman, 2007; Rauch et al., 2008). Further work is needed to better understand what aspect of neural activity is reflected by BOLD signals in cases where there is dissociation between LFPs and the BOLD signal, as occurs in the hippocampus (as discussed in Ekstrom, 2010).

The vast majority of fMRI studies describe only the properties of BOLD signals and make only qualitative inferences of their implications for the underlying neural activity. Conversely, in electrical and magnetic forms of non-invasive brain imaging the recorded signal derives directly from the functional activity of neurons (though with varying degrees of transmission from the neural origin to the scalp recording sites), but the ability to localize these signals with any degree of accuracy remains remarkably elusive as the complexity of brain activation for even the simplest of tasks tends to confound attempts to resolve the local neural components contributing to the recorded scalp responses. To improve our estimates of neural signals using non-invasive brain imaging techniques, this Frontiers Research Topic invited empirical and theoretical contributions focusing on the explicit relationship of brain imaging signals to causative neural activity.

The submitted contributions responded to the challenge of neural signal estimation in a variety of ways including: advanced analyses of the neural implications of magnetoencephalographic (MEG) and electroencephalographic (EEG) signals, derivations of the pathway for BOLD signal generation from the underlying neural activation signals through animal recording, human BOLD modeling studies, detailed assessment of local BOLD response components and resting-state activation, and interpretation of the new field of functional diffusion tensor imaging in terms of neural activation.

Although the EEG and MEG commonly used to measure human neural activity have high temporal resolution, spatial localization of the signal source is difficult to achieve. Cicmil et al. highlighted the limits on localizing MEG signal sources by testing the ability of several reconstruction approaches to localize the source of retinotopic MEG signals in the human brain and found that none of the approaches for assessing angular position were suitable for resolving annular stimuli spanning different retinal eccentricities (unless restricted in angular position). A second contribution to such electrical signal analysis is the time-frequency approach to the source localization and functional connectivity from simultaneous MEG/EEG signals proposed by Zerouali et al. Although, this analysis specifically targeted sleep spindles, the work has broader implications for the functional integration of MEG and EEG signals and their source localization within the brain. This analysis revealed that functional connectivity across the cortex evolved during the spindles from short-range intra-hemispheric connections to longer range inter-hemispheric connections, suggesting an integrative role for these dynamic features of neural activity.

Several contributions focused on estimating the properties of the underlying neural sources that generate BOLD fMRI signals. Martin reviews the need for accurate neurovascular models of the coupling between neural activity and the local BOLD signal from animal studies. Animal studies have the striking advantage of allowing a wide variety of technical approaches to the analysis of neurovascular coupling. Martin evaluates 16 of these, from single-neuron electrophysiology to tissue oxygen voltammetry, considering both their advantages and limitations and highlighting the key areas in which our understanding of fMRI signals has been improved through the use of animal models. Howarth takes up the issue of whether cortical astrocytes (glial cells), and calcium transients within them, are involved in the vascular response to neuronal activity based on the recent debate regarding whether evoked glial calcium signals occur quickly enough to account for the dynamics of neurovascular coupling. Indeed, the exact mechanisms by which astrocytes respond to changes in neuronal activity and trigger the intracellular events regulating the resulting vascular response underlying the fMRI BOLD signal remain unclear. To take an analytic approach to this question, Tyler et al. evaluate four models for the neurovascular coupling between local field potentials recorded in cortex and BOLD signals recorded simultaneously in an adjacent location, for a range of stimulus durations. The results imply that the BOLD response is most closely coupled with metabolic demand derived from the neuronal input waveform, suggesting that the astrocytic signaling is responsive to the neurotransmitter metabolism of the dendritic arborization rather than to the neuron's spiking activity.

Further studies focus on contributions to the positive and negative components of the neurovascular relationships. Buxton et al. assess the coupling ratio of blood flow and oxygen metabolism to different kinds of neural activation, finding that blood flow variations are more closely coupled with stimulus-driven variations than with endogenous variations in neural activity (e.g., those driven by attention, adaptation, and generalized excitability). Variations in oxygen metabolism, on the other hand, are more closely coupled with endogenous neural variations. The authors suggest that these differences in coupling ratio reflect differential proportions of excitatory and inhibitory contributions of the neural signal to cortical BOLD signals, and hence provide a new window into the assessment of neural activity. A related topic is addressed by Chen, who uses stimulus-driven manipulations of activation and suppression to assess the excitatory and inhibitory contributions to the evoked BOLD signal. The stimuli were designed to have invariant local effects, but differential long-range interactions were found according to configural relationships of local orientations, which should produce no differences in BOLD signal in the absence of neural interactions. One component of the BOLD suppression was dependent on the orientation-specific inhibitory effect of the long-range interactions, while a second appeared to be a general negative BOLD response to adjacent contrast stimulation independent of the stimulus configuration. Thus, BOLD response properties can be used to identify targeted aspects of the underlying neural organization.

Three papers focus on advanced methods of decomposing the neural connectivity and reorganization in the brain from the distribution of BOLD signals. Gonzalez-Castillo et al. take the novel approach of analyzing the time-course of resting-state BOLD signals across the cortex to assess the stability of neural connectivity. The most stable connections were between homologous (symmetric) interhemispheric local regions, with stability persisting for several minutes. The more variable connections were found to correspond primarily to occipito-frontal connections across the traditional resting-state networks, which can be interpreted as corresponding to transient visual imagery. Gravel et al. take resting-state analysis a step further to develop the concept of local cortical *connective* fields. These are neural organizations analogous to neuronal receptive fields, but defined in terms of connectivity among cortical regions, rather than connectivity of the neuron to a sensory surface. In combination with the population receptive mapping developed by this group for the analysis of the visual cortex, resting-state BOLD connectivity can be interpreted in visual space. This approach allows visuotopic maps to be reconstructed using resting state data recorded in the visual cortex, enabling these authors to show that the local resting-state connectivity from visual area V1 to both V2 and V3 was invariant with eccentricity with a scale of ~2 mm, substantially smaller than the population receptive fields for visual input in these cortical areas. This work suggests that it is possible to obtain some neural properties from resting-state fMRI data.

Concentrating on the example of motor learning, Yang et al. extend the analysis of BOLD activation maps. Learning may generate not only changes in the strength of activation in predefined regions of interest, but also changes in the spatial distribution of the activation across the cortex. To address this issue, the authors measure the changes in spatial distribution of activation following a simple motor learning task. Dimension reduction via singular-value decomposition was able to capture aspects of the neural reorganization produced by this form of motor learning. These findings validate the capability of computational modeling to determine properties of neural connectivity and reorganization from BOLD signal analysis.

The final two papers are concerned with a new *functional* form of Diffusion Tensor Imaging (DTI). DTI is a well-established technique for assessing the anatomical organization of the fiber pathways *in vivo* from the local anisotropy of the diffusion directions of water molecules within brain tissue. Functional DTI, on the other hand, assesses *changes* in this kind of anisotropy as a result of some functional manipulation of the state of the brain. Autio and Roberts raise concerns about contamination of this form of functional analysis by leakage of BOLD signal activation from adjacent gray matter into the voxels designated as fiber pathways. Mandl et al., whose previous paper on functional changes in fractional anisotropy in the optic radiations during visual stimulation was the subject of the Autio and Roberts critique, argue that such partial voluming would only occur at the ends of fiber tracts where they meet with the cortical regions that they are connecting, whereas the reported changes in fractional anisotropy occurred throughout the tracts.

In summary, functional imaging techniques are increasingly used to infer neural activity within the human brain. This special issue improves our ability to estimate these neural signals non-invasively and points us in the direction of the remaining issues that must be addressed before we can fully understand functional imaging signals.

## Author contributions

All authors listed, have made substantial, direct, and intellectual contribution to the work, and approved it for publication.

## Funding

CH was a Vice Chancellor's Advanced Fellow at the University of Sheffield and currently holds a Sir Henry Dale Fellowship jointly funded by the Wellcome Trust and the Royal Society (grant number:105586/Z/14/Z).

### Conflict of interest statement

The authors declare that the research was conducted in the absence of any commercial or financial relationships that could be construed as a potential conflict of interest.
